# Mitchell–Riley Syndrome: Improving Clinical Outcomes and Searching for Functional Impact of RFX-6 Mutations

**DOI:** 10.3389/fendo.2022.802351

**Published:** 2022-06-22

**Authors:** Caroline de Gouveia Buff Passone, Gaëlle Vermillac, Willem Staels, Alix Besancon, Dulanjalee Kariyawasam, Cécile Godot, Cécile Lambe, Cécile Talbotec, Muriel Girard, Christophe Chardot, Laureline Berteloot, Taymme Hachem, Alexandre Lapillonne, Amélie Poidvin, Caroline Storey, Mathieu Neve, Cosmina Stan, Emmanuelle Dugelay, Anne-Laure Fauret-Amsellem, Yline Capri, Hélène Cavé, Marina Ybarra, Vikash Chandra, Raphaël Scharfmann, Elise Bismuth, Michel Polak, Jean Claude Carel, Bénédicte Pigneur, Jacques Beltrand

**Affiliations:** ^1^ Department of Endocrinology, Metabolism and Diabetes, Inserm U1016, Cochin Institute, Paris, France; ^2^ Pediatric Endocrinology, Gynecology and Diabetology, Centre de Référence des Pathologies Gynécologiques Rares et des Maladies Endocriniennes Rares de la Croissance et du Développement, Hôpital Universitaire Necker Enfants Malades, Université Paris Descartes, Paris, France; ^3^ Beta Cell Neogenesis (BENE) Research Group, Vrije Universiteit Brussel (VUB), Brussels, Belgium; ^4^ Division of Pediatric Endocrinology, Department of Pediatrics, Universitair Ziekenhuis Brussel (UZ Brussel), Vrije Universiteit Brussel (VUB), Brussels, Belgium; ^5^ Imagine Institute, Hôpital Universitaire Necker Enfants Malades, Université Paris Descartes, Paris, France; ^6^ Pediatric Gastroentherology Hepatology and Nutrition Unit, Hôpital Universitaire Necker Enfants Malades, Université Paris Descartes, Paris, France; ^7^ INSERM UMR S 1139, Faculté de Pharmacie de Paris, Université de Paris, Paris, France; ^8^ Hepatology Unit, Hôpital Universitaire Necker Enfants Malades, Université de Paris, Inserm U1151, Centre de Référence Maladie rares Atresie des voies biliaires et cholestases génétiques et Filière de soin Filfoie, Paris, France; ^9^ Pediatric Surgery Department, Hôpital Universitaire Necker Enfants Malades, Université Paris Descartes, Paris, France; ^10^ Pediatric Radiology Department, Hôpital Universitaire Necker Enfants Malades, Université Paris Descartes, Paris, France/INSERM U1163, Institut Imagine, Paris, France; ^11^ Neonatal Intensive Care Unit, Hôpital Universitaire Necker Enfants Malades, EHU 7328 Université Paris Descartes, Paris, France; ^12^ Université Paris Cité, Hôpital Universitaire Robert-Debré, Service d’Endocrinologie Diabétologie Pédiatrique et CRMR Prisis, Paris, France; ^13^ Pediatric Department Hôpital d’Enfants de Margency Croix-Rouge française, Margency, France; ^14^ Department of Pediatric Gastroenterology and Nutrition, Hôpital Universitaire Robert-Debré, Paris, France; ^15^ Genetic Department, Hopital Universitaire Robert Debré, Paris, France; ^16^ Research Center of Sainte Justine University Hospital, Université de Montréal, Montreal, QC, Canada; ^17^ Biomedicum Stem Cell Center, Faculty of Medicine, University of Helsinki, Helsinki, Finland

**Keywords:** neonatal diabetes mellitus, RFX6, Mitchell–Riley syndrome, diabetes technology, beta-cell function, parenteral nutrition

## Abstract

**Aims/Hypothesis:**

Caused by biallelic mutations of the gene encoding the transcription factor *RFX6*, the rare Mitchell–Riley syndrome (MRS) comprises neonatal diabetes, pancreatic hypoplasia, gallbladder agenesis or hypoplasia, duodenal atresia, and severe chronic diarrhea. So far, sixteen cases have been reported, all with a poor prognosis. This study discusses the multidisciplinary intensive clinical management of 4 new cases of MRS that survived over the first 2 years of life. Moreover, it demonstrates how the mutations impair the *RFX6* function.

**Methods:**

Clinical records were analyzed and described in detail. The functional impact of two RFX6^R181W^ and RFX6^V506G^ variants was assessed by measuring their ability to transactivate insulin transcription and genes that encode the L-type calcium channels required for normal pancreatic beta-cell function.

**Results:**

All four patients were small for gestational age (SGA) and prenatally diagnosed with duodenal atresia. They presented with neonatal diabetes early in life and were treated with intravenous insulin therapy before switching to subcutaneous insulin pump therapy. All patients faced recurrent hypoglycemic episodes, exacerbated when parenteral nutrition (PN) was disconnected. A sensor-augmented insulin pump therapy with a predictive low-glucose suspension system was installed with good results. One patient had a homozygous c.1517T>G (p.Val506Gly) mutation, two patients had a homozygous p.Arg181Trp mutation, and one patient presented with new compound heterozygosity. The RFX6^V506G^ and RFX6^R181W^ mutations failed to transactivate the expression of insulin and genes that encode L-type calcium channel subunits required for normal pancreatic beta-cell function.

**Conclusions/Interpretation:**

Multidisciplinary and intensive disease management improved the clinical outcomes in four patients with MRS, including adjustment of parenteral/oral nutrition progression and advanced diabetes technologies. A better understanding of *RFX6* function, in both intestine and pancreas cells, may break ground in new therapies, particularly regarding the use of drugs that modulate the enteroendocrine system.

## Introduction

Mitchell–Riley syndrome (MRS; OMIM #615710) is a rare disease characterized by neonatal diabetes, pancreatic hypoplasia, gallbladder agenesis or hypoplasia, and duodenal atresia (1, 2). It is caused by biallelic mutations in the regulatory factor X6 gene (RFX6) located on chromosome 6q22 (2). *RFX6* expression was demonstrated in embryonic precursors of pancreatic islet cells, and its transcription factor is important to the normal development and differentiation of insulin-secreting beta-cells (2–4). *RFX6* expression begins early in life throughout the anterior endoderm and, at later stages, seems to be restricted to the gut and pancreas endocrine cells (2, 5, 6). Furthermore, *RFX6* knockout causes the intestinal cells to show reduced expression, production, and secretion of the glucose-dependent insulinotropic polypeptide (GIP), leading to reduced insulin response (6, 7). *RFX6* is a transcriptional activator of *insulin* gene (INS) expression and is required for normal Ca^2+^ channel activity supportive of depolarization-evoked insulin secretion in human beta-cells (4). The disease is heterogeneous probably due to the different types of mutations involving RFX6 (7, 8).

Clinical management can be difficult, as neonatal diabetes in MRS is associated with severe digestive and hepatic dysfunction (8, 9). Low birth weight, protracted diarrhea, and persisting feeding difficulties related to bowel surgery contribute to failure to thrive (7, 8). Other abnormalities such as cholestasis, hepatic failure, heterotopic pancreatic tissue, anemia, thrombocytopenia, hemochromatosis, hypospadias, clotting, and metabolic disorders (acidosis) were described in MRS, but their physiopathology is not yet completely understood (9). So far, sixteen cases have been reported, caused by homozygous (n = 12) or compound heterozygous mutation (n = 4) in the *RFX6* (9, 10). Of the 12 homozygous cases, seven died of sepsis and liver failure before the age of 6 months, and data on clinical status after 1 year of age are scarce (8, 9).

We aim to report the multidisciplinary and intensive clinical management and outcomes of four new MRS cases that survived longer than 2 years of age and to demonstrate the functional loss of the mutated *RFX6* protein caused by the homozygous mutations.

## Methods

We report four new MRS cases with confirmed variants in *RFX6.* Two cases were genetically confirmed in the neonatal period. Patients 1, 3, and 4 were followed up at the Necker Enfants Malades University Hospital and patient 2 at the Robert Debré University Hospital, both situated in Paris, France. All information was collected from clinical records in both hospitals between 2008 and 2021. The patients’ parents provided written informed consent, and the study was conducted in accordance with institutional guidelines.

### Genetic Testing

Patients and their parents had the *RFX6* (NM_ 173560.3) sequenced in a panel of neonatal diabetes mellitus of 24 genes by capture-based next-generation sequencing (NGS) on genomic DNA extracted from peripheral blood. NGS of each gene (all the exons identified in NCBI RefSeq+/− 50 intronic flanking bp) was performed onto a NextSeq500R (Illumina, San Diego, CA, USA) after Custom SureSelectQXT enrichment (Agilent, Santa Clara, CA, USA). Image analysis, alignment of sequence reads to the reference human genome build GRCh37 (hg19), and variant calling were performed on the BaseSpaceR (Illumina) by using Burrows-Wheeler Aligner (BWA) enrichment v2.1 pipeline (Illumina). Variants were classified by employing Bench Lab NGS, v5.0.2 (Cartagenia) according to the American College of Medical Genetics and Genomics (ACMG) recommendations, by making use of population databases (GnomAD), databases of variant interpretation (ClinVar, HGMD professional-QIAGEN), and computational *in silico* prediction tools (PolyPhen2, SIFT, CADD) (11).

### Functional Impact of *In Vitro* Analysis

In order to evaluate the functional impact of the RFX6 mutation, we assessed the ability of two of the RFX6 variants (R181W and V506G) to transactivate the insulin promoter and to induce the expression of the genes that encode subunits of L-type calcium channels (*CACNA1A*, *CACNA1C*, *CACNA1D*, and proton-sensing ion channels GPR68) required for normal pancreatic beta-cell function. Additionally, we used Human Embryonic Kidney 293-cell line (HEK cells) to evaluate if these mutations could modify the nuclear location of RFX-6 gene.

### Culture of Cell Lines

The human beta-cell line EndoC-βH2 was cultured as previously reported (12) in low-glucose (5.6 mM) Dulbecco’s modified Eagle’s medium (DMEM; Sigma-Aldrich, St. Louis, MO, USA) containing l-glutamine and sodium pyruvate, supplemented with 2% bovine serum albumin (BSA) fraction V (Roche Diagnostics, Basel, Switzerland), 50 µM of 2-mercaptoethanol, 10 mM of nicotinamide (Calbiochem), 5.5 µg/ml of transferrin (Sigma-Aldrich), 6.7 ng/ml of selenite (Sigma-Aldrich), 100 U/ml of penicillin, and 100 µg/ml of streptomycin. Cells were seeded at a density of 5 × 10^4^ cells/cm^2^ on Matrigel (1%)/fibronectin (2 µg/ml) (Sigma-Aldrich) coated plates and cultured at 37°C and 5% CO_2_. HEK-293 cells were cultured in high-glucose DMEM supplemented with 10% fetal calf serum (Biowest, Nuaillé, France) and 100 U/ml of penicillin and 100 µg/ml of streptomycin.

### *RFX6* Constructs and DNA Transfection

Human *RFX6* constructs (pCDNA3.1-wtT-RFX6; pCDNA3.1-MutV506G-RFX6; plasmid Polylinker Retroviral IRES GFP (pRIG)-VP16 wtRFX6-IERS-EGFP; pRIG-VP16MutV506G-RFX6-IERS-EGFP) was previously described (4). The *RFX6* mutant (C541T; p.R181W) was constructed in pCDNA3.1-wtT-RFX6 and pRIG-VP16 wtRFX6-IERS-EGFP by using the Q5 Site-Directed mutagenesis kit (NEB). The position of the mutation was confirmed by DNA sequencing. EndoC-βH2 cells were transiently transfected with DNA constructs by using Lipofectamine 2000 (Invitrogen, Carlsbad, CA, USA) according to the manufacturer’s instructions in Opti-MEM.

### Human Insulin Promoter Analysis, Constructs, and Luciferase Reporter Assays

The human insulin promoter constructs pGL2 human-INS (-378 to +42), kindly provided by Dr. Melloul (Hadassah University Hospital, Jerusalem, Israel), was subcloned into the basic firefly reporter vector pGL4.12[luc2CP] (Promega, Madison, WI, USA) (7). For transactivation of insulin promoter-driven luciferase reporter assay, EndoC-βH2 cells were co-transfected by using Lipofectamine 2000 with INS promoter-driven luciferase reporter, pcDNA3.1(+) empty or RFX6^WT^ or RFX6^V506G^ or RFX6^R181W^ vector and Renilla luciferase. Luciferase activities were measured 24 h later. Firefly and Renilla luciferase activities were measured with a Dual-Luciferase reporter assay system (Promega) according to the manufacturer’s instructions. Firefly luciferase activities were normalized to Renilla luciferase activities to correct for the transfection efficiency.

### RNA Isolation, Reverse Transcription, and Quantitative RT-PCR

Total RNA was extracted from EndoC-βH2 cells by using the RNeasy Micro kit (Qiagen, Hilden, Germany). First-strand cDNA was prepared by employing the Maxima First Strand cDNA synthesis kit (Thermo Fisher, Waltham, MA, USA). Real-time PCR was performed using Power SYBR Green mix (Applied Biosystems, Foster City, CA, USA) with ABI Prism 7300 sequence detector (Applied Biosystems). Cyclophilin A transcript levels were used for the normalization of each target gene. The custom primers were designed with IDT Primer-Quest online software, and the amplification efficiency for each primer was determined with serial dilution of total cDNA from EndoC-βH2 cells (4).

### Immunocytochemistry

HEK-293 cells were transfected with pRIG empty vector or pRIG-wtRFX6 or pRIG-MutR181W or pRIG-MutV506G constructs using Lipofectamine 2000 (Invitrogen) following manufacturer’s instructions in Opti-MEM. Post 48 h of transfection cells were processed for immunostaining as described previously (4) with rabbit anti-RFX6 primary antibody (Sigma-Aldrich #HPA037697, 1:200 dilution) and Texas red anti-guinea pig secondary antibody (Jackson ImmunoResearch Laboratories, West Grove, PA, USA; 1:500 dilution).

### Statistics

Quantitative data are presented as the mean ± SEM from at least three independent experiments, unless specified otherwise. Statistical significance was calculated using an unpaired two-tailed Student’s t-test. Statistical significance was set at p < 0.05.

## Results

### Description of the Patients’ Cases

Clinical characteristics are described in **Table 1**. All patients had a prenatal diagnosis of duodenal atresia. The main clinical features at birth were that they were all born small for gestational age, had a duodenal malformation, and developed early hyperglycemia. All patients underwent surgery during the first week of life. The parents of the three patients with homozygous RFX6 mutations were consanguineous, but the parents of the patient with compound heterozygosity were not. There was no family history of diabetes.

**Table 1:** Clinical characteristics of four reported patients.

Case number	1	2	3	4
Genetic mutation	c.1517T>G, (p.Val506Gly)	c.541 C>T (p.Arg181Trp)	c.541 C>T (p.Arg181Trp)	c.505-2A>G et c.2782A>G
Sex	F	M	F	M
Consanguinity	Yes	Yes, type 2 diabetes in second-degree relatives	Yes	No
Origin	Caribbean (Martinique Island)	French (gypsy community)	Portuguese and Spanish	French and Spanish
Gestational age (weeks)/birth weight (g)/birth length (cm)	35/1,390/no information	37 + 4/1,290/38	36+1/1,765/43	37+4/1,984/44
Intestinal atresia	Duodenum and jejunum	Duodenum	Duodenum	Triple atresia (two in duodenum, one in jejunum)
Gut malrotation	No	No	Yes	Yes
Histology abnormalities	Meckel’s diverticulum	No	Meckel’s diverticulum, ectopic pancreatic tissue	Meckel’s diverticulum. Ectopic gastric tissue in duodenum
Chronic diarrhea	++	++++	++++	++++
PN dependency index^*^ last evaluation	No PN (stopped at 1 year old)	82%	58%	119%
Enteral nutrition a day	No need for enteral nutrition	2 bolus of 22 ml/kg each through gastrostomy	No need for enteral nutrition	No enteral nutrition due to no tolerance
Feeding disorders	No	Yes	No	Yes
Pancreatic anomaly	Global hypoplasia	Hypoplastic tail and agenesis of the body	Global hypoplasia	Annular pancreas
Pancreatic enzyme replacement	Yes, for a year, not effective	Yes, not effective	Yes, partial effect	Yes, partial effect
Cholestatic diseases	No	Yes, for 1.5 years	Yes, 2–7 months old	Yes, from day 1 to liver transplant
Liver failure	No	No	No	Yes, liver transplant at 7 months old
Anemia/treatment	Yes/RBC transfusion and IV iron supplementation	Yes/RBC transfusion and IV iron supplementation	Yes/repeated RBC transfusions	Yes/RBC transfusions
Other clinical features	G6PD deficiency	Pica disorder	Persistent acidosis	–
	Epiphyseal dysplasia	Intermittent acidosis		
		Flat nails and brittle teeth		
Motor development	Adequate	Walked at 20 months	Small delay	Small delay
			Walked at 20 months	Walked at 14 months
Age at last evaluation	13 years 6 months	7 years 2 months	2 years 6 months	2 years 3 months

RBC, red blood cells; PN, parenteral nutrition.

^*^PN dependency index: ratio of non-protein-energy intake provided by PN for achieving normal or catching up body weight gain.

**Figure 1:**
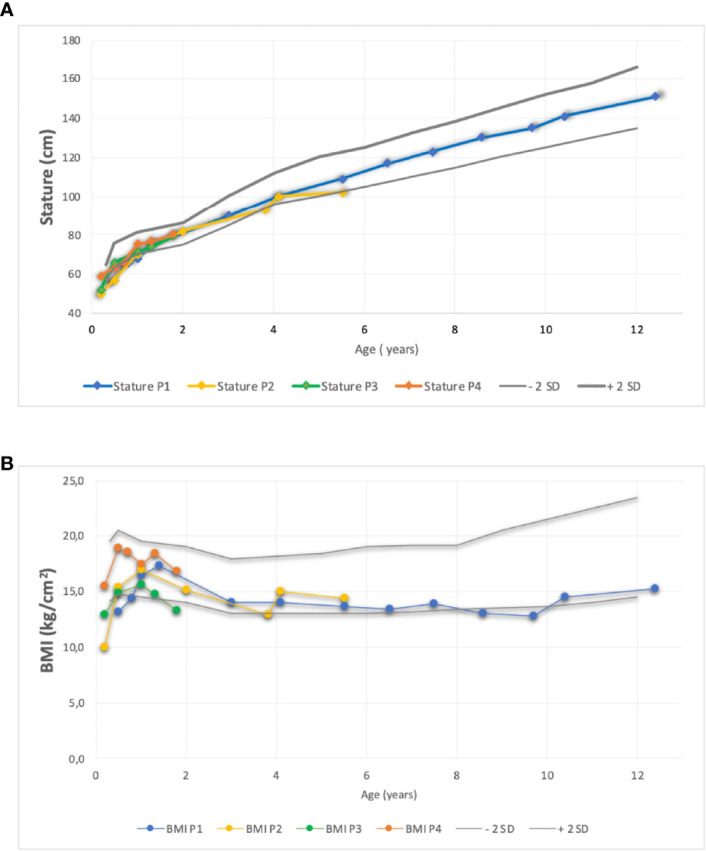
Comparison of stature **(A)** and **(B)** Body Mass Index evolution of four patient using standard deviation scores according to WHO growth charts. BMI, Body M ass Index; P, patient, SD, Score Deviation.

All patients failed to thrive during the first month of life. They all received nutritional support and effective antibiotic treatment for invasive infections. Their height was between −2 and 1 SD (**Figure 1**), and their body mass index (BMI) was between −1.5 and −3.0 SD. Patients 2, 3, and 4 had some degree of delayed motor development (see **Table 1**).

**Figure 2:**
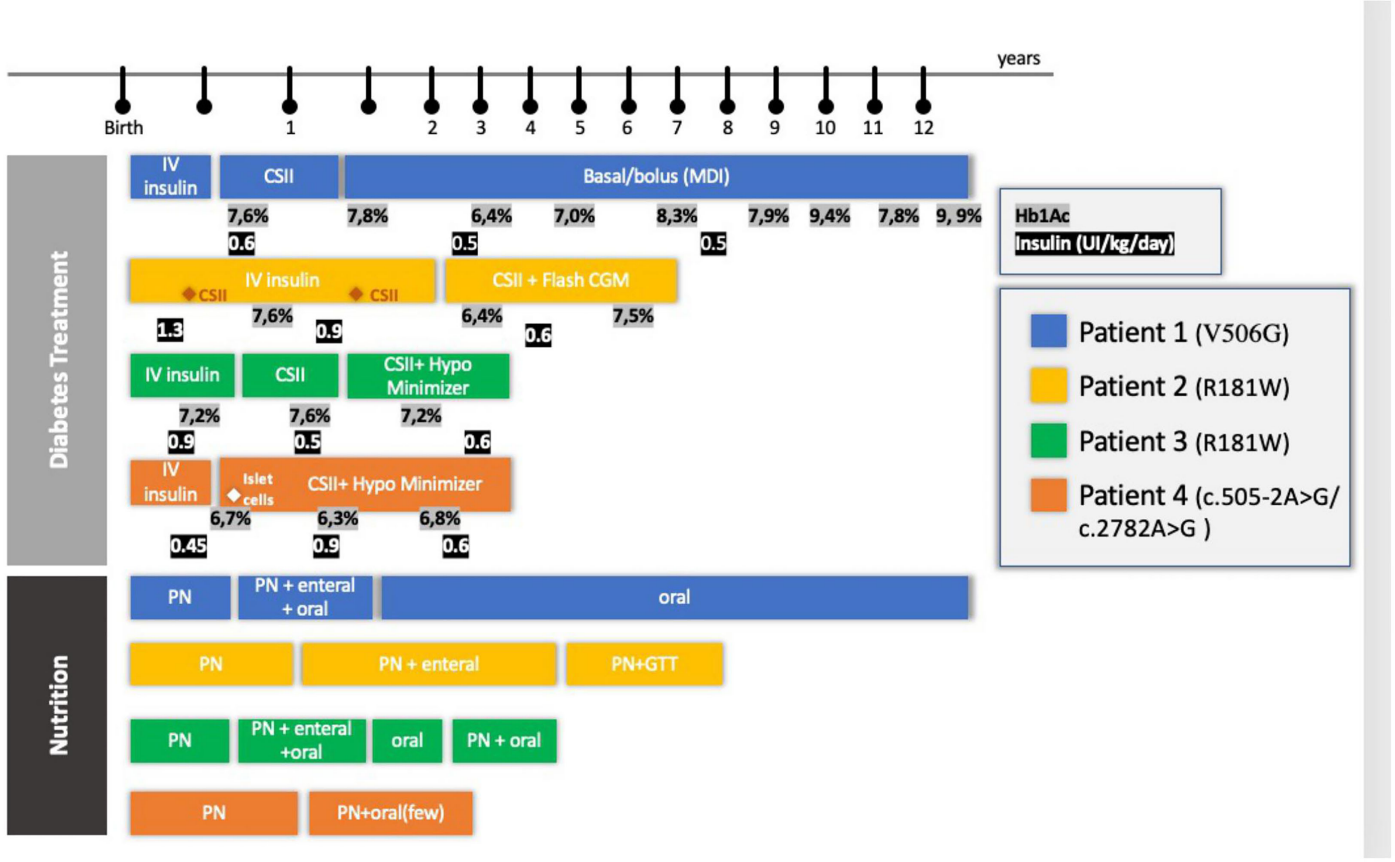
Diabetes Treatment and Nutrition follow-up of four patients with RFX-6 mutation. PN, parenteral nutrition; CSII, Continuous Subcutaneous Infusion System; Hb1Ac, glycosylated hemoglobin; CGM, continuous monitoring system; GTT, gastrostomy; HypoMinimyzer, The Predictive Hypoglycemia Minimizer System.

Follow-up management according to each area of interest will be described below. Diabetes management and nutrition progression are described in **Figure 2**.

#### Diabetes Management

All patients received routine blood sugar monitoring due to prematurity and low birth weight and presented with hyperglycemia (between 300 and 500 mg/dl) on the first or second day of life. Neonatal diabetes was diagnosed in the first week of life, and intravenous (IV) insulin treatment was installed. Insulin was adjusted by the endocrinology team in collaboration with the neonatology and the gastroenterology team, with daily doses ranging from 0.6 to 1.3 IU/kg. All cases presented with low C-peptide.

Recurrent non-ketotic hypoglycemic episodes frequently occurred and were exacerbated by episodes of sepsis, metabolic disturbances, acidosis, and liver dysfunction, but no severe hypoglycemic event was reported. The response to oral/enteral glucose supplementation was impaired (patients needed 20–30 g of oral glucose), and glucagon was relatively ineffective. The hypoglycemic episodes were reverted with IV glucose supplementation (10% glucose, 2 ml/kg). Later, with the disconnection of parenteral nutrition (PN) and the introduction of enteral nutrition, severe intestinal malabsorption was likewise implicated. These hypoglycemic episodes were difficult to treat, as there was a limited response to oral/enteral glucose supplementation (twice the usual dose of oral sugar was needed) and relative ineffectiveness of glucagon. Only IV glucose supplementation at the usual dose was effective. In those cases, to avoid hypoglycemia, for patients in continuous subcutaneous insulin infusion (CSII), the basal rate was decreased or suppressed. On the other hand, during PN therapy, sustained hyperglycemia also occurred, and the basal rate had to be increased more than 10 times in this period. Aside from that, occasionally, a prolonged bolus was programmed to match the carbohydrates and the fat contained in these solutions.

Patient 1, female, started CSII at 3 months of age and switched to multiple daily injections (MDI) when she was 1.5 years old. This switch was required by the family to allow more freedom during playtime. Her HbA1c levels were fairly stable during early childhood (between 48 (6.5%) and 59 mmol/mol (7.5%)]. Although she still had hypoglycemic episodes, her HbA1c levels increased at the outset of puberty from 62 (7.8%) to 84 mmol/mol (9.9%). Currently, she is 13 years old and on an MDI schedule with fixed doses of glargine and aspart insulin.

Patient 2, male, failed to transition to CSII at 2 and 5 months of age because of sepsis episodes and high glycemic variability. At 2 years of age, his HbA1c was 40 mmol/mol (5.8%). An insulin pump was introduced and programmed with a low basal rate (0 to 0.025 UI/h) throughout the period he was disconnected from PN. From then onward, he used a flash continuous glucose monitoring (CGM) system and obtained moderate glycemic control. At 3 years of age, he had two acute diabetic ketoacidosis episodes. All attempts made to restart enteral feeding resulted in glycemic decompensation and diarrhea. At the age of 4 years, he received two insulin boluses when fed in addition to basal insulin infusion.

**Figure 3:**
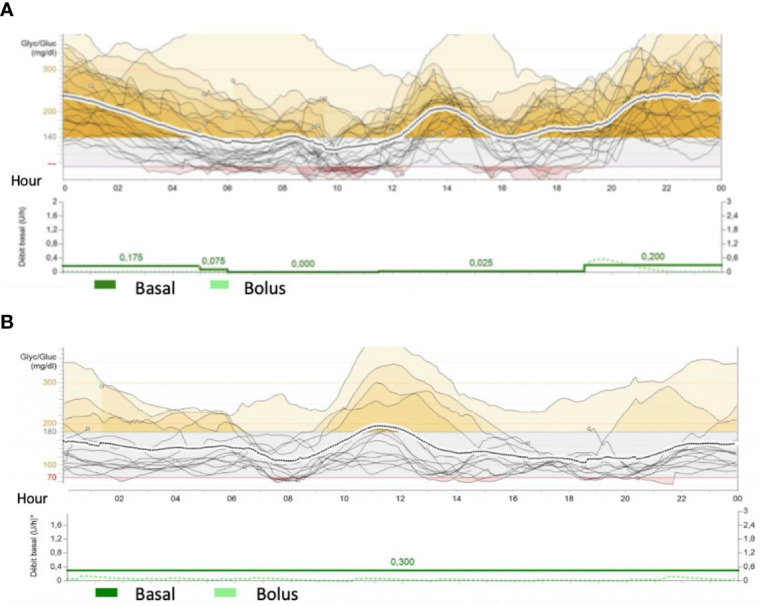
Conti nous Glucose Monitoring da ta from Mini Med 640G System from patients 3- M utR181W **(A)** and 4- (c.505-2A>G/ c.2782A>G ) **(B)**. The traces represents glucose variability during the day. Red colour cshowed hypoglycemic periods (less than 70mg/dl) and yellow periods represents hyperglycemias ( more than 140-180 mg/dl) In figure **(A)**, patient 3 presents recurrent hypoglycemic episodes during daily period when she was disconnect from parenteral nutrition (4 a.m.) and frequent hyperglycemia during night period. Basal rate was 0.2UI of insulin during parenteral nutrition (around 10 times more than during the day) Patient 4 had lower glycemic variability, but some hypoglycemia du ring the day. He received parenteral nutrition and few oral nutrition.

Patient 3, female, transitioned to CSII only at 5 months of age due to recurrent infections. To avoid recurrent hypoglycemia throughout the period, she was disconnected from PN, and a predictive low-glucose suspension system was successfully installed. She was discharged at 7 months of age with CSII and a predictive hypoglycemia minimizer (**Figure 3**). At 2 years of age, her insulin doses were between 0.4 and 0.9 IU/kg/day, divided into 3 boluses (lunch, 4 p.m. snacks, and when PN starts) with good metabolic control [highest HbA1c, 55 mmol/mol (7.2%)].

Patient 4, male, had good metabolic control and transitioned to CSII with a predictive hypoglycemia minimizer at 4 months old. His mean glycemia in the sensor was 150 ± 68 mg/dl, and his estimated HbA1c was 59 mmol/mol (7.6%). At the age of 7 months, he progressed to an end-stage liver disease and was referred for a liver transplant. This created the opportunity to perform a joint beta-cell transplant with a chance of improving diabetes control in the short and medium term. Eight days after his liver transplantation, he received islet-cell transplantation: approximately 700,000 suspended islets isolated from the same donor as the liver graft were injected into the spleen. Unfortunately, C peptide levels remained low at 0.12nmol/L (normal range, 0.25–1.20 nmol/L) and insulin requirements did not decrease, indicating failure of islet-cell transplantation. Glycemic control was managed throughout the transplantation period and thereafter maintained by using CSII with a predictive hypoglycemia minimizer (**Figure 3**). At the last follow-up meeting, at 2 years of age, he was taking 1 bolus of insulin at the outset of PN and had a high basal rate throughout his PN infusion (0.65 UI/kg between 7 p.m. and 7 a.m.).

#### Gastrointestinal Abnormality Management

All patients underwent abdominal surgery for correction of the duodenal atresia early in life. PN was crucial to support weight gain and prevent diarrhea. To avoid cholestasis, many attempts to start minimal enteral nutrition were made, but all patients developed profuse diarrhea, weight loss, and abdominal distention. Amino acid-based formula was tried without improvement.

Patient 1 underwent abdominal surgery at 11 days of life where all atretic intestine was resected. Exclusive PN was maintained for 3 months, and afterward, oral feeding was gradually introduced. She was discharged from the hospital at 11 months of age with combined oral feeding and cyclic nocturnal PN infusion for 12 h. PN was discontinued 2 months later. At her 13-year-old checkup, she had good intestinal transit, with 4 daily stools and no rectal blood or mucus loss. She remained with postprandial urge bowel incontinence.

**Figure 4:**
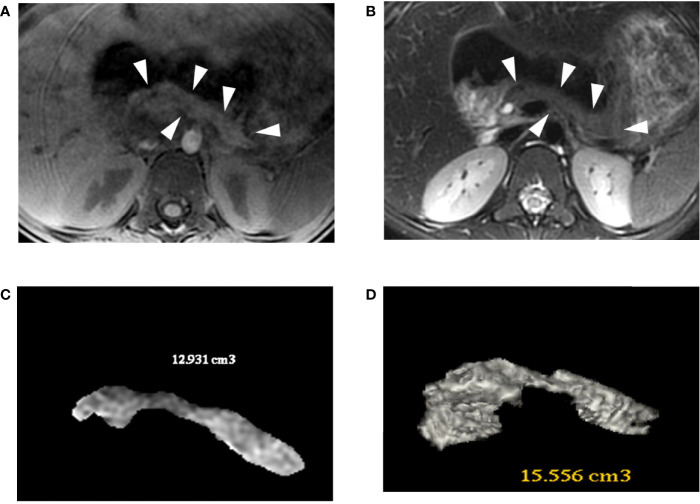
Patient 2 (R181W) 3D-MRJ T2 **(A)** and Tl-weighted **(B)** with volume rendering reconstructions **(C, D)**. The MRI shows a small pancreas (white arrowheads), especially in the bod y and tail, with a volume estimated between 13 and 15.5 cc. For comparison, an aged-matched control patient has a pancreatic volume of 45 cc.

Patient 2 underwent surgery for duodenal atresia correction on day 3 of life. Abdominal MRI showed a pancreatic tail and body agenesis, gallbladder agenesis, and a normal biliary tract (**Figure 4**). Enteral nutrition was progressively introduced, but after several failed attempts, exclusive PN was maintained. Simultaneously, he developed a pica eating disorder. He was hospitalized at 3.8 years old with an acute intestinal obstruction complicated with ulcerations and gastritis, where a large piece of plastic was found in the duodenum. At 4 years of age, enteral nutrition was restarted and associated with cyclic PN infusion. At 5.6 years old, a gastrostomy was performed to maximize his enteral feeding since oral feeding was not evolving favorably.

Patient 3 underwent surgical repair of the duodenal atresia on day 1 of life and had a short period of exclusive PN. At 5 months of age, a cow milk protein exclusion diet allowed an expansion of oral feeds and a progressive decrease in PN infusion to 14 h per day. She was able to sustain oral feeding from 22 to 26 months of life but had to restart PN due to prolonged weight loss after an acute gastroenteritis episode.

Patient 4 had a Ladd’s procedure performed on the first day of life in association with duodenal atresia correction, appendectomy, and Meckel’s diverticulum resection. A tracheoesophageal fistula was found and treated by laser therapy on day 9. He was on exclusive PN for 3 months when partial breast milk feeding was introduced, followed by complementary food with good oral tolerance. At 6.5 months of age, an endoscopy revealed ectopic gastric tissue on a fragment of the duodenum. He was discharged at 13 months old, with continuous PN infusion, no enteral nutrition, and few oral feedings due to severe malabsorption. At 19 months old, he was on cyclic PN for 16 h per day and restarted oral feeding (1 or 2 tablespoons of mashed fruit per day).

Patients 1 and 2 also presented with rectal bleeding and elevated cow milk protein IgE levels (>1.5 kUA/L, reference range <0.3 kUA/L) and had a diagnosis of IgE-mediated cow milk protein allergy (CPMA). Cow milk protein was successfully reintroduced at 2 years of age in patient 2. Patients 1 and 3 suffered from necrotizing enterocolitis at ~2 months of age and were treated with antibiotics and discontinuation of enteral feeding for 3 weeks.

In all patients, exocrine pancreatic insufficiency was suspected in the third week of life due to steatorrhea and/or low levels of elastase (patient 2, 183 μg/g; patient 3, <15 μg/g; patient 4, 15 μg/g; normal range >200 μg/g). Patients 1 and 2 were treated with pancreatic enzyme replacement therapy during the first year of life, but no beneficial effects were observed. Patients 3 and 4 are still under pancreatic enzyme replacement therapy (approximately 5,000 UI/kg/day), but they maintained steatorrhea (lipid in stool, 6 g/24 h, normal range 0.5–3 g/24 h). Fecal elastase levels increased in patient 3 but did not normalize (144 μg/g of stool).

#### Liver and Gallbladder Disease Management

Patient 1 had no liver problems. Patient 2 presented with cholestasis at 11 days of life and at 1.4 years old, with hepatic cytolysis. Both episodes normalized after treatment with ursodeoxycholic acid. Patient 3 presented with mild cholestasis from birth to 7 months of age but never required treatment. Patient 4 presented neonatal cholestasis and then liver failure and received a liver transplant when he was 7 months old.

#### Anemia Management

All patients presented normocytic normochromic anemia since birth. They received multiple blood transfusions and iron infusions during their first year of life.

#### Others

Three patients (2, 3, and 4) had persistent acidosis requiring bicarbonate replacement in different periods of their lives.

All patients presented with vitamin K deficiency. Patients 1 and 2 presented intermittent deficiency, corrected by intravenous supplementation, with normalization and no recurrence. Patients 3 and 4 received oral and IV vitamin K supplementation but remained with low FVII levels (between 15% and 30%; reference FVII > 50%); congenital FVII deficiency was excluded.

All patients had multiple infections associated with a long hospitalization period. Extended-spectrum beta-lactamase (ESBL) producing enterobacteria; coagulase-negative staphylococci (CoNS) (coagulase-negative staphylococcus) bacteria were found in all of them. Each patient presented at least one catheter-related sepsis requiring catheter replacement.

### Genetics and Description of Functional *In Vitro* Studies

**Figure 5:**
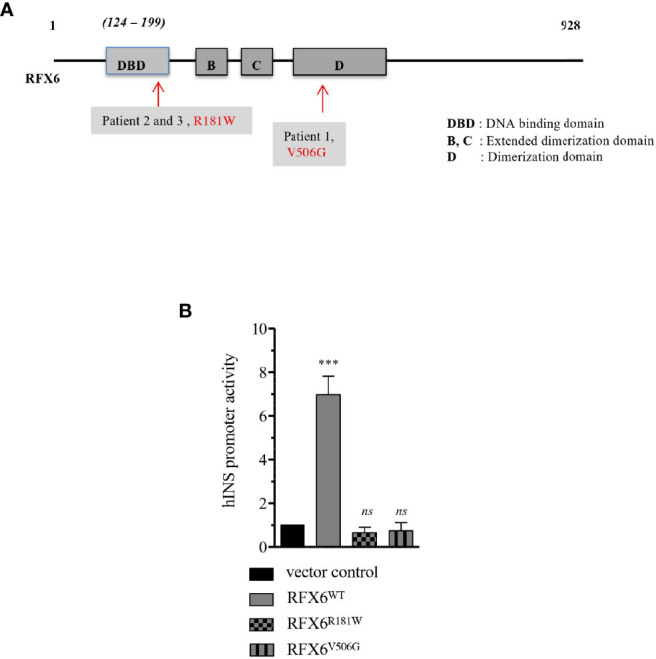
Transactivation of Insulin promoter by RFX6^WT^, RFX6^R181W^ and RFX6^V506G^ in EndoC-βH2 cells. **(A)** Schematic presentation of the functional domains in RFX6 protein and the position of mutations. **(B)** Insulin promoter activity in human beta cells model EndoC-βH2 cells determined by firefly luciferase (pGL4.12hu*INS*-378to+42), which was cotransfected with either RFX6^WT^ or RFX6^R181W^ or RFX6^V506G^ and with *Renilla* luciferase (pGL4.72-TK[hRlucCP]) to correct for variation in transfection efficiency. Results are presented as fold increase over empty control vector. Da ta are mean of ± SEM of three to four experiments. ***p < 0.001; *ns*, *non significant*.

The homozygous c.1517T>G (p.Val506Gly) mutation variant found in patient 1 had been previously reported but with a limited description of its clinical characteristics (7). Patients 2 and 3 had a homozygous c.541C>T (p.Arg181Trp) mutation, which showed heterozygous in both healthy parents. This variant as well as another substitution targeting the same amino acid had been previously reported in patients with MRS (Amorim 2015; Smith 2010). It targets a highly conserved amino acid within the *RFX6* DNA-binding domain (**Figure 5A**). Patient 4 had a compound heterozygous for a paternally c.505-2A>G and maternally c.2782A>G p.(Thr928Ala) inherited variants. *In silico* predictions were in favor of a splice effect (loss of an acceptor site) for the c.505-2A>G variant. The c.2782A>G variant has unknown significance based on CADD (24.5 > 23.4), polyphen 2 (benign), and SIFT (low impact), and it has been reported once in GnomAD (no clinical data). Pathogenicity is mainly inferred from its rarity, together with a biparental inheritance of *RFX6* variants and convincing clinical features. In addition, no other variant was found by testing a panel of the 24 most frequent genes involved in neonatal diabetes.

### Functional *In Vitro* Results

To evaluate the ability of RFX6 variants to transactivate the insulin promoter, we constructed separate expression plasmids for each of the following variants: RFX6^WT^ as the wild-type control, RFX6^R181W^ for the variant in patients 2 and 3, and RFX6^V506G^ (7) for the variant in patient 1. Each construct was co-transfected into cells of the human beta-cell line EndoC-βH2 with a plasmid containing the immediate promotor region of INS, driving a luciferase reporter.

The RFX6^WT^ construct induced strong activation of the reporter, while the responses to the RFX6^R181W^ and RFX6^V506G^ variants were not significantly different from those induced by an empty expression vector control (**Figure 5B**). In conclusion, these 2 mutations resulted in lower hINS promoter activity.

**Figure 6:**
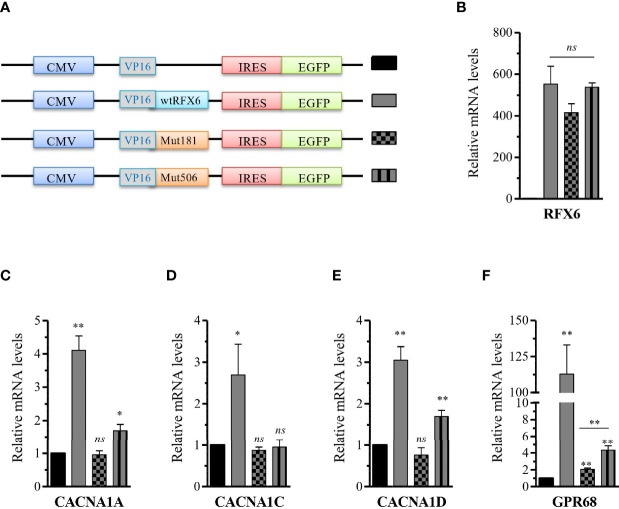
Differential activation of R FX6 targets gene expression by wtRFX6 or Mu tR181W-R FX 6 or MutV506G-RFX6 in EndoC-βH2 cells. **(A)** Schematic presentation of the constructs with trans-activation domain VP16 and I ERS-EGFP used to overexpress wt or Mut R FX6 proteins **(B) **Fort y-eight hours posttransfection, GFP+ cells were FACS sorted and analyzed for the expression of R FX6 by RTq PCR in EndoC-βH2. **(C–F)** RT qPCR analysis of CACNAlA, CACNA1C, CACNA1D and G PR 68 in EndoC-βH2 post 48h transfected with wt or Mut RFX6. Da ta are mean of ± SEM of three to four experiments. *p < 0.05; **p <0.01; ns, non significant.

Next, in order to characterize the functional impact of the RFX6^R181W^ and RFX6^V506G^ variants, we constructed new expression plasmids, in which the variants wtRFX6, MutR181W-RFX6, and MutV506GRFX6 were preceded by a promiscuous VP16 activation domain and linked with an internal ribosome entry site (IRES) to a fluorescent eGFP reporter allowing assessment of the 5′ variant expression level (**Figure 6A**). Each construct was again transfected into EndoC-βH2 cells, and relative *RFX6* expression upon transfection was found to be similar in each of the conditions (**Figure 6B**). To examine the functional consequences of these RFX6 variants, we assessed the ability of these *RFX6* variants containing plasmids to activate known downstream targets. We found that wtRFX6 strongly induced several genes encoding subunits of L-type calcium channels including *CACNA1A*, *CACNA1C*, *CACNA1D*, and proton-sensing ion channels GPR68 (13). The MutV506G-RFX6 variant induced only a mild response for *CACNA1D* and *GPR68*, whereas for the other genes, the response was not significantly different from that induced by empty expression vector control. This absence of any response was also the case for the MutR181W-RFX6 for all tested genes (**Figures 6C–F**). These findings suggest that MutV506G-RFX6 and MutR181W-RFX6 are unable to induce the expression of the calcium channel genes required for normal pancreatic beta-cell function.

**Figure 7:**
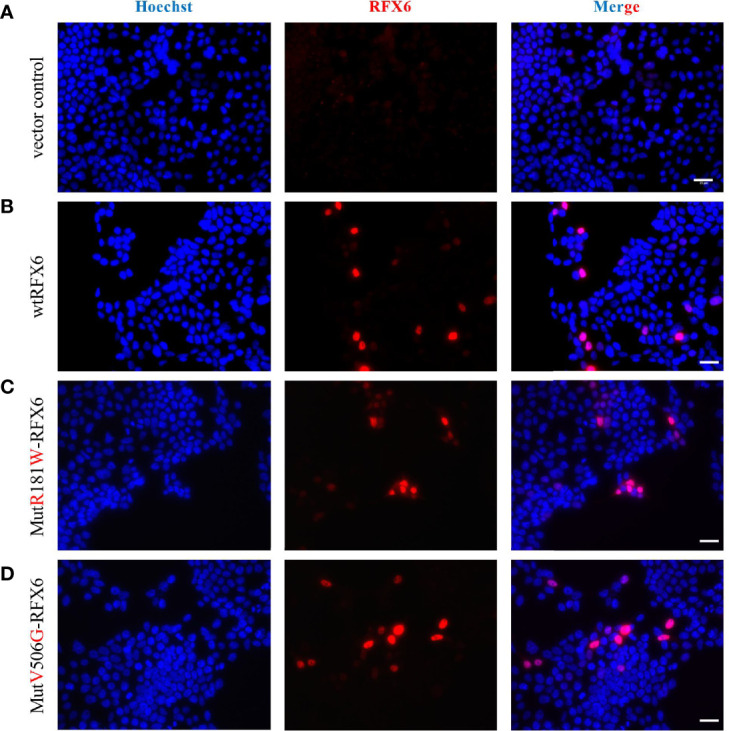
Expression and localization of of wtRFX6 or MutR181W-RFX6 or MutV506G-RFX6 in HEK293. HEK293 cells were transiently transfected with pRIG-empty or pRIG-wtRFX6 or pRIG-MutR181W or pRIG­MutV506G -R FX6 constructs and immunostained for RFX6 post 48 h of transfection. Scale bar 25 μm. Legend: **(A)** Vector control, **(B)** wtRFX6, **(C)** Mut R181W-RFX6, **(D)** MutV506G-RFX6.

In addition, we assessed the behavior of these variants in the Human Embryonic Kidney 293-cell line, hereafter called HEK. HEK cells do not express endogenous RFX6 and are therefore entirely responsive to heterologous expression. HEK cells transfected with the above-described plasmids were used to assess the intracellular location of each variant. Cells transduced with either wtRFX6, MutR181W-RFX6, and MutV506G-RFX6 are all appropriately located in the nucleus itself (**Figures 7A–D**). That means that despite the mutation, such a location is still preserved (**Figures 7A–D**).

## Discussion

We report the disease course of four genetically confirmed cases of MRS. In line with previous studies, neonatal diabetes was the key to making the diagnosis in a newborn with duodenal atresia (1, 8, 9, 14–17).

In the first 2 years of life, to obtain better glycemic control, our patients required CSII with a predicted low-glucose suspension system due to recurrent hypoglycemia. This system facilitated the adjustment to enteral nutrition attempts, avoiding episodes of hypoglycemia during fasting and food bolus, and hyperglycemia during a long period of PN. We hypothesized that hypoglycemia was mainly related to exogenous insulin administration and their impaired response to oral supplementation. The rapidity of its onset was possibly related to some residual endogenous secretion. It has been suggested that these patients have irregular glucagon secretion, which also contributes to hypoglycemia (18).

Our patients suffered from profuse diarrhea and difficulties thriving. PN was necessary to maintain weight gain and growth in the first year of life and sometimes for longer periods. Early attempts to wean off PN induced glycemic decompensation. Enteral nutrition was carefully introduced with a slow increase and close monitoring of intestinal tolerance (stool–frequency and volume, abdominal distension, pain, and vomiting). To decrease complications, especially liver-related ones, it was important to respect the gut physiology by discontinuing PN and stimulating the oral–gut axis. In addition, stimulating oral and enteral feeding was crucial to prevent feeding disorders, as well as to stimulate the gastrointestinal tract and enterohepatic axis.

Pancreatic enzyme replacement therapy is still controversial in the literature. Some authors support that despite the reduced pancreatic size, there is no pancreatic enzyme deficiency, and therefore patients would be unresponsive to supplementation (2, 8, 9, 14, 15). Evidence also includes two autopsies, revealing normal-appearing exocrine pancreas with clusters of chromogranin-A-positive cells and adequate gene expression by knockout mice (2, 8). On the other hand, pancreatic abnormalities are heterogeneous depending on the type of mutation involved. Pearl and collaborators described in 2011 a loss of exocrine gene expression in an *in vitro* experiment using a *Xenopus RFX6* (5, 14). All our patients received pancreatic enzyme replacement therapy at some point in their care, with partial response. There was no change in diabetes control associated with this therapy. It has been recently proposed to focus on the enteroendocrine cell dysfunction and to use GLP-1 analogs for the management of protracted diarrhea and diabetes in patients with heterozygous *RFX6* mutations (16, 18). Detailed reports of the diarrhea are important for underlying molecular genetic mechanisms (6, 8, 18).

It is important to monitor for cholestasis and liver function in the neonatal and in the follow-up periods when patients are under NP. In total, three out of our four cases presented with intrahepatic cholestasis, and only six patients were similarly reported in the literature (8, 9). In two of our patients, the cholestasis was intermittent, self-limited, and only required a short period of treatment with ursodeoxycholic acid. In one patient though, it led to liver failure requiring liver transplantation, as previously described by Concepcion and colleagues (8).

Severe anemia has been described in seven other reported cases (17). Our patients were all born anemic and benefited from several red blood cell (RBC) transfusions in their first year of life. Skopkova et al. postulated that the anemia was secondary to gastric mucosa heterotopy, thus leading to inflamed gut mucosa and bleeding (17); however, only two of our patients presented with it. Although anemia was noticed in the first week of life, none of our patients had bloody stools as previously reported by Conception et al. (8) and Khan et al. (8, 9). PN iron deficiency could explain part of it, but the full mechanism implicated in the severe anemia is still unknown. The multiple blood transfusions to treat the severe anemia could have also compromised the evaluation of HbA1c in our patients.

Mutation c.541 C>T (p.(Arg181Trp), as found in patients 2 and 3, was described in the same residue by Pearl et al. (p.R181Q) (5) and Zegre Amorim et al. (p.R181W) (19). All had consanguineous parents and intestinal atresia, received multiple RBC transfusions, and showed mild cholestasis. Some of them developed ectopic pancreatic tissue with dependence on PN at between 60% and 82%. Our patients also had the same clinical characteristics but did not develop ectopic pancreatic tissue.

Our group had earlier reported an RFX6^V506G^ variant, showing *INS* expression reduction and secretion due to reduced transactivation of genes encoding subunits of L-type calcium channels (4). Similarly, in this study, we generated and tested the RFX6^R181W^ variant for the potential to activate insulin and other downstream targets of *RFX6*. Our data showed that mutant RFX6^R181W^ does not interfere with the expression or location of the RFX6 protein but has failed to activate the INS promoter or X-box containing RFX6 targets gene P/Q, L-type calcium channels, and *GPR68* required for normal pancreatic beta-cell function. The arginine at the codon position 181 in the DNA binding domain of *RFX6* is highly conserved across species, and it is very likely to affect the protein function, whose subject has to be further explored (5). On the other hand, the protein is located in the nucleus, as expected (**Figures 7A–D**). The normal subcellular location of RFX6 is in the nucleus. Detection of RFX-6 in the cytoplasm might have indicated nucleocytoplasmic shuttling as a mechanism explaining the differences in the transcriptional response to wild-type or mutant RFX6. A recent article also showed that *RFX6* is essential for efficient differentiation of pancreatic endoderm, whose absence in individuals with MRS specifically impairs the formation of endocrine cells of the pancreas head and tail (20). Ultimately, *RFX6* gene plays an important functional and developmental role in beta-cells (21).

Strengths of this study include that although the use of IV insulin in patients with MRS has been previously reported, few had data on glycemic control, and none had described the importance of hypoglycemia treatment and the use of CSII with a predictive hypoglycemia minimizer (1, 8, 9). We also described the first beta-cell transplant in an infant with RFX-6 mutation and the oldest patient (13 years old) with MRS homozygous mutation. Furthermore, the functional analysis impact of these mutations paves the way for new therapeutic approaches. The limitations of this study include a lack of functional analyses of the *RFX6* compound heterozygous mutations and the unexplained mechanism related to severe anemia.

## Conclusions

In patients with intestinal atresia, gallbladder agenesis, and neonatal diabetes, RFX6 mutation should be considered.

Multidisciplinary and intensive management of patients with MRS can significantly improve clinical outcomes. They can benefit from the adjustment of parenteral/oral nutrition progression in association with sensor-augmented pump therapy with a predictive hypoglycemia minimizer system.

If PN is needed for the first year of life, stimulating the oral and gut axis is important to allow nutrition evolution and prevent complications.

There is a negative functional impact of the R181W and V506G variants in transactivating the INS promoter and inducing the expression of the genes that encode subunits of L-type calcium channels.

A better understanding of *RFX6* function, in both intestine and pancreas cells, may lead to new therapies, particularly regarding drugs that modulate the enteroendocrine system.

## Data Availability Statement

The raw data supporting the conclusions of this article will be made available by the authors, without undue reservation.

## Ethics Statement

Written informed consent was obtained from the individual(s), and minor(s)’ legal guardian/next of kin, for the publication of any potentially identifiable images or data included in this article.

## Author Contributions

CP and GV wrote the original draft. AB, DK, CG, CL, CT, EB, MN, CaS, AP, CoS, ED, and TH treated the patients. A-LF-A, YC, and HC carried out the genetic analysis. LB produced the image. VC and WS conducted the molecular studies. CC, MG, BP, MP, JC, EB, AL, RS, MY, and JB supervised the writing. All authors helped review the article with the aim of adding important intellectual content and provided the final approval.

## Funding

This study was funded by ANR Blanc RFX-PancInt (RS) and the LABEX Revive (RS). WS holds a senior clinical investigator grant from the Research Foundation Flanders (File number: 77833).

## Conflict of Interest

The authors declare that the research was conducted in the absence of any commercial or financial relationships that could be construed as a potential conflict of interest.

## Publisher’s Note

All claims expressed in this article are solely those of the authors and do not necessarily represent those of their affiliated organizations, or those of the publisher, the editors and the reviewers. Any product that may be evaluated in this article, or claim that may be made by its manufacturer, is not guaranteed or endorsed by the publisher.
